# CRISPRi-mediated repression of three *cI* repressors induces the expression of three related *Neisseria gonorrhoeae* bacteriophages

**DOI:** 10.1128/jb.00049-25

**Published:** 2025-05-12

**Authors:** Wendy E. Geslewitz, H Steven Seifert

**Affiliations:** 1 Department of Microbiology and Immunology, Northwestern University 3270, Chicago, Illinois, USA; University of California San Francisco, San Francisco, California, USA

**Keywords:** *Neisseria gonorrhoeae*, bacteriophage, CRISPRi

## Abstract

The *Neisseria gonorrhoeae* FA1090 isolate encodes nine prophage islands (Ngoɸ1–9). Ngoɸ1–3 contain genes consistent with a *Siphoviridae*-dsDNA bacteriophage (phage). Saturating transposon-sequencing screens using two different *N. gonorrhoeae* isolates predicted that multiple prophage genes were essential, including three putative transcriptional repressors: *ngo0479* (present in Ngoɸ1), *ngo1116* (present in Ngoɸ2), and *ngo1630* (present in Ngoɸ3). All three genes display homology to the Lambda phage *cI*, a regulator important for maintaining the lysogenic state and inhibiting lytic induction, but these proteins are not close paralogs. Using a *Neisseria lactamica*-derived Type I-C CRISPR-interference system, we show that these *cI* orthologs are essential, as the knockdown of each gene results in bacterial death. We determined that the repression of the three *cI* orthologs resulted in the significant induction of phage gene expression. Finally, we detected *Siphoviridae*-like phage particles released from *N. gonorrhoeae* following repression of *ngo0479*, *ngo1116*, or *ngo1630*. We hypothesize that these *cI* orthologs are critical for preventing phage lytic infection and cell death and allow *N. gonorrhoeae* to benefit from the carriage and expression of prophage genes.

**IMPORTANCE:**

Bacteriophage, or phage, are bacteria-infecting viruses and are the most abundant natural entities in the world. Here, we report that *Neisseria gonorrhoeae*’s three most complete double-stranded DNA prophage islands each encode essential and related transcriptional repressors. CRISPRi-mediated repression of these transcriptional repressors leads to a significant increase in prophage gene expression and phage induction. This study marks an important initial step in studying the interaction between *N. gonorrhoeae* and its resident phage.

## INTRODUCTION

Gonorrhea is the second most prevalent sexually transmitted infection, with over 600,000 new cases in the United States in 2023 (1). *Neisseria gonorrhoeae* (Gc, gonococcus) is a gram-negative, human-restricted pathogen and the most common etiological agent of gonorrhea infection. Gc commonly colonizes mucosal xtissues, including the urogenital tract (2), nasopharyngeal tissue (3), and the ocular (4) and anal mucosa (5). Gc’s multiple antigenic variation systems allow it to alter the expression and composition of its surface structures, making it adept at evading the immune system (6–9). As such, infection elicits an unproductive immune response that does not protect against subsequent infections (10, 11). Furthermore, Gc has grown increasingly resistant to antibiotics, with recent isolates showing resistance to front-line antibiotics (12–14). Further investigation into Gc’s biology is needed for a better understanding of this organism.

Initial work has been performed to characterize Gc phage. *In silico* analyses determined that the genome of the Gc isolate FA1090 possesses nine prophage islands; four islands (Ngoɸ6–9) encode filamentous, single-stranded DNA phage, and five islands (Ngoɸ1–5) encode predicted tailed, double-stranded DNA phage (15, 16). Gc’s filamentous islands display homology to the *N. meningitidis* filamentous MDAɸ phage, the best-characterized phage species in the *Neisseria* genus (17, 18). When the genome of Ngoɸ6 was inserted into a plasmid and cloned into different bacterial species, these species were able to produce filamentous phage particles, suggesting that this island may produce phage in Gc (19). The tailed phages of the *Caudoviricetes* class are the most abundant and diverse bacteriophage (20). Saturating transposon-sequencing studies performed with two different Gc isolates found that several phage-related genes were unhit by a transposon (21; Hu and Seifert, unpublished data). These studies suggest that these genes may be essential.

Previous studies have also found that Gc prophage genes could contribute to critical bacterial functions. Anaerobic growth induced the expression of 47 prophage genes within islands Ngoɸ1–3 and Ngoɸ5, suggesting that these islands may be important for growth in the reproductive tract’s low oxygen conditions or biofilm formation (22). Indeed, 20 prophage genes displayed differential expression in Gc isolated from the genital tract of gonorrhea-infected women compared to bacteria grown in growth media (23). Furthermore, in response to treatment with sublethal levels of hydrogen peroxide, a critical component of neutrophil-mediated antimicrobial defense, 18 Gc prophage genes were induced (24). Interestingly, an insertional mutation in Ngoɸ4’s Fur-responsive transcriptional repressor Npr resulted in the enhanced ability of bacteria to infect endothelial cells and colonize mice (25). Collectively, these data suggest that Gc’s prophage genes play a role in its adaptation to relevant physiological conditions.

While there has not been a direct demonstration of Gc phage production, there are indirect data showing that some of these phage islands may produce active phage virions. A bioinformatic analysis examining almost 4,000 unique CRISPR-Cas targeting spacers from the *Neisseria* genus found that targeting spacers specific to the Gc prophage islands were enriched compared to those targeting the bacterial chromosomal backbone (26). This result suggests that other *Neisseria* species may have previously interacted with Gc phage. However, it is unclear whether phage can be produced from Gc.

Phages often persist in the host DNA as prophages, only to revert to the lytic cycle in response to external stimuli (27–29). Critical for preventing this switch is the CI transcriptional repressor, a regulator that represses transcription of the lytic genes and inhibits phage production. Initially identified in Lambda phage, CI orthologs are found in many phage species (30, 31). CI proteins can undergo proteolysis through interaction with the activated RecA filament formed by replication fork stalling following DNA damage (32). This mechanism of CI degradation facilitates the production of phage particles and bacterial lysis in conditions where it is more favorable for phage to exist separately from the host.

Here, we identified three CI orthologs (NGO0479, NGO1116, and NGO1630) encoded in Gc’s three more complete phage islands (Ngoɸ1, Ngoɸ2, and Ngoɸ3). We show that these genes are essential, as repression of all three genes with CRISPR interference resulted in bacterial death (33). RNA sequencing revealed that repression of each gene significantly increased phage gene expression from Ngoɸ1–3. Finally, we could visualize phage particles released following repression of each transcriptional repressor.

## RESULTS

### Gc encodes three paralogous dsDNA prophage islands

**Fig 1:**
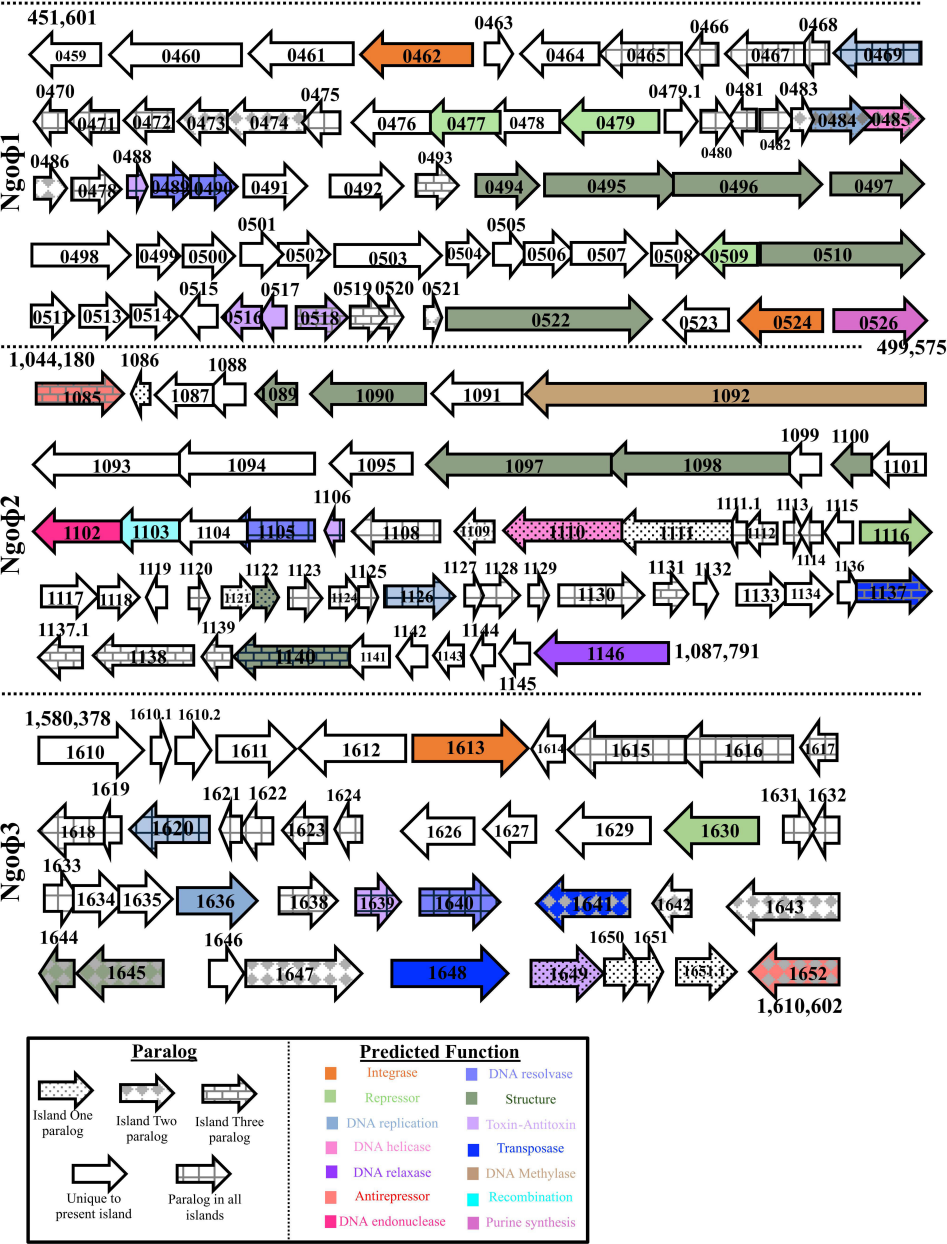
Map of dsDNA prophage islands Ngoɸ1, Ngoɸ2, and Ngoɸ3. Arrows represent prophage genes. The shape pattern indicates whether the gene is unique to the island or paralogous to genes in the other prophage islands. The shape color indicates the predicted function of the gene based on its amino acid sequence. A white arrow signifies that the gene has no predicted function.

We examined the five predicted FA1090 dsDNA prophage islands to assess the intactness of each phage. We used a previous *in silico* analysis and the computational tool Phaster, which predicts dsDNA prophage islands within bacterial genomes, to predict the completeness of each phage island (34, 35). The smallest islands are Ngoɸ4 and Ngoɸ5, which only contain 17 and 11 Open Reading Frames (ORFs), respectively (15). Phaster designated Ngoɸ4 as incomplete while failing to identify Ngoɸ5 as a prophage (15). We focused our analysis on islands Ngoɸ1, Ngoɸ2, and Ngoɸ3, which Phaster predicts may be functional phage islands (Fig. 1).

Ngoɸ1, Ngoɸ2, and Ngoɸ3 contain significant sequence homology to each other, but there are also ORFs unique to each island. Ngoɸ1 is the largest island, at a length of ~48 kB and comprising 67 genes (Table S2). Ngoɸ2 is the next largest, at ~43 kB and 62 genes, followed by Ngoɸ3 at ~30 kB and 44 genes. The filamentous prophage islands Ngoɸ6 (7.1 kB, 11 ORFs) and Ngoɸ9 (7.2 kB, 8 ORFs) were predicted as being part of NGOɸ2 and NGOɸ3, so we did not differentiate them from the dsDNA islands (15).

There are 50 paralogous genes shared between the three islands (Fig. S1). Ngoɸ1 encodes the highest number of unique, non-paralogous genes at 36, followed by Ngoɸ2 with 32 and Ngoɸ3 with 17. A higher proportion of the genes is conserved between Ngoɸ2 and Ngoɸ3. Almost 16% of genes in Ngoɸ3 are paralogous to genes encoded in Ngoɸ2, and 11% of Ngoɸ2’s genes have paralogs in Ngoɸ3. Alternatively, only 4.5% of Ngoɸ1’s genes have paralogs in Ngoɸ3 and 10% in Ngoɸ2. Thus, while these islands share many paralogs, each has differences.

Each phage island also contains genes predicted to encode dsDNA phage structural and regulatory functions. Within Ngoɸ1, there are five genes with predicted structural functions: *ngo0494* (terminase small subunit), *ngo0495* (terminase large subunit), *ngo0496* (phage portal protein), *ngo0497* (protease structural protein), *ngo0510* (phage tail protein), and *ngo0522* (tail length tape measure protein). Ngoɸ2 encodes five predicted dsDNA structural proteins: *ngo1089* (phage structural protein), *ngo1090* (phage structural protein), *ngo1097* (phage portal protein), *ngo1098* (phage terminase large subunit), and *ngo1100* (terminase small subunit). Ngoɸ3 does not contain any genes with predicted structural functions. Each island also encodes genes with predicted DNA replication and packaging functions, such as *ngo0469*/*ngo1130*/*ngo1620* (replication initiation), *ngo0484*/*ngo1111*/*ngo1636* (phage DNA replication protein), *ngo0485*/*ngo1110* (DnaB helicase), and *ngo0489*/*ngo0490*/*ngo1105*/*ngo1640* (RusA endodeoxyribonuclease). Each island also encodes a predicted holin gene (*ngo488*/*ngo1106*/*ngo1639*); holins puncture holes in the bacterial membrane to facilitate lysis and phage escape from the host (36). This *in silico* analysis supports the hypothesis that the NGOɸ1–3 islands may produce phage.

### *ngo0479*, *ngo1116*, and *ngo1630* are *cI* orthologs

**Fig 2:**
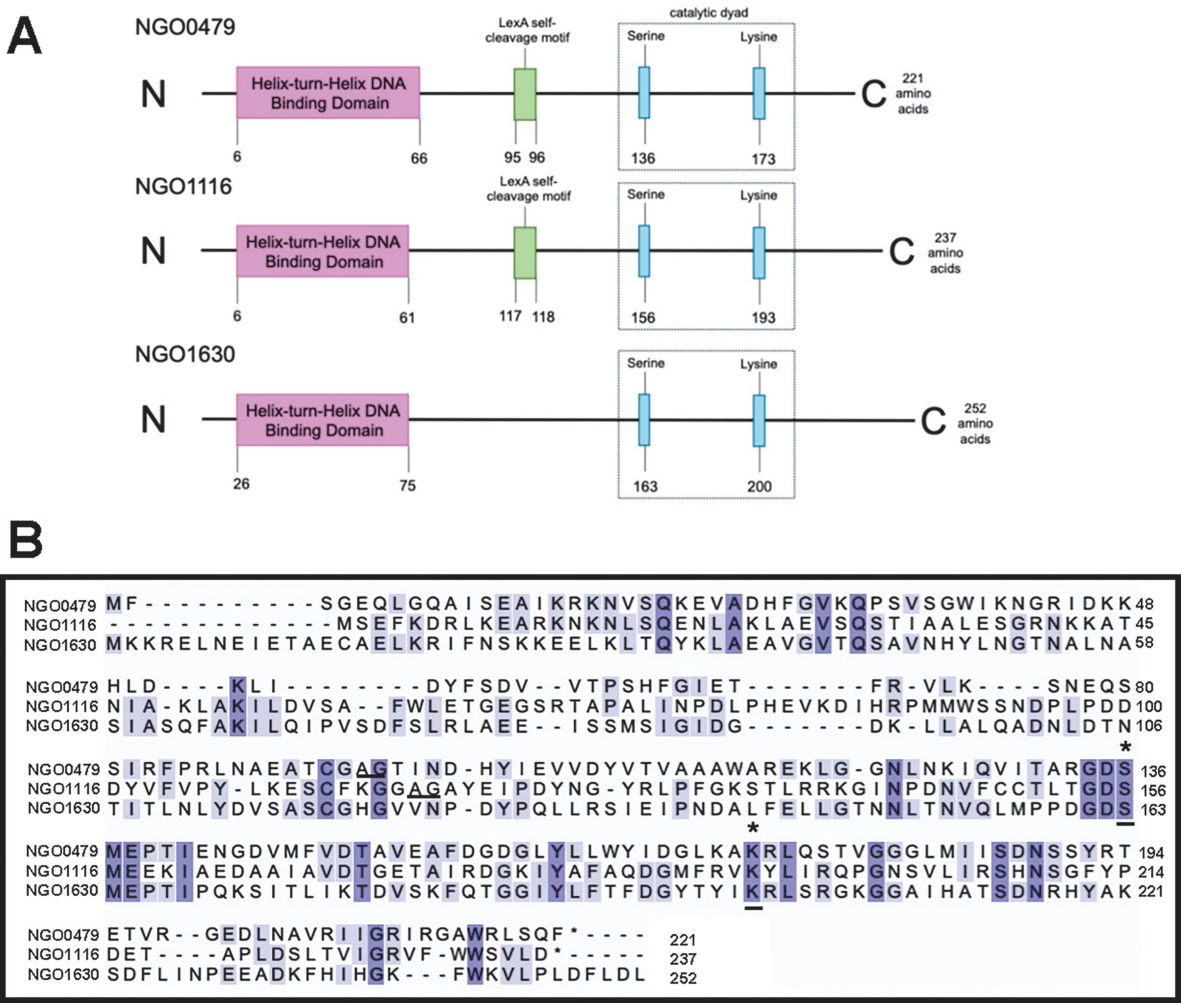
NGO0479, NGO1116, and NGO1630 are CI orthologs. (**A**) A schematic of predicted CI-like domains within the predicted NGO0479, NGO1116, and NGO1630 proteins. Each domain is marked with a black line and a number corresponding to the residue in the protein sequence. (**B**) Clustal-Omega multiple sequence alignments of NGO0479, NGO1116, and NGO1630 proteins. Amino acids highlighted in dark indigo indicate identical residues among all three proteins, while lighter indigo highlights indicate identical residues among two proteins. Black underline indicates conserved, CI-like residues; asterisks are annotated above the serine-lysine catalytic dyad motif.

Each of the three Gc’s dsDNA phage islands encodes a predicted *cI* ortholog type repressor: *ngo0479* (Ngoɸ1), *ngo1116* (Ngoɸ2), and *ngo1630* (Ngoɸ3) (Fig. 2A). The predicted proteins range in size from 221 to 252 amino acids, and each contains an N-terminus helix-turn-helix DNA-binding domain and a C-terminus S24 serine-lysine peptidase. NGO0479 and NGO1116—but not NGO1630—each encode the predicted alanine-glycine self-cleavage motif, which is characteristic of CI proteins (37). Despite sharing similar predicted domains, a Clustal-Omega sequence alignment indicates these three proteins are only weak paralogs (Fig. 2B) (38). NGO0479 shares 19.4% identity with NGO1116 and 24.0% identity with NGO1630, while NGO116 and NGO1630 share 25.6% identity.

### All three *cI* orthologs are essential

**Fig 3:**
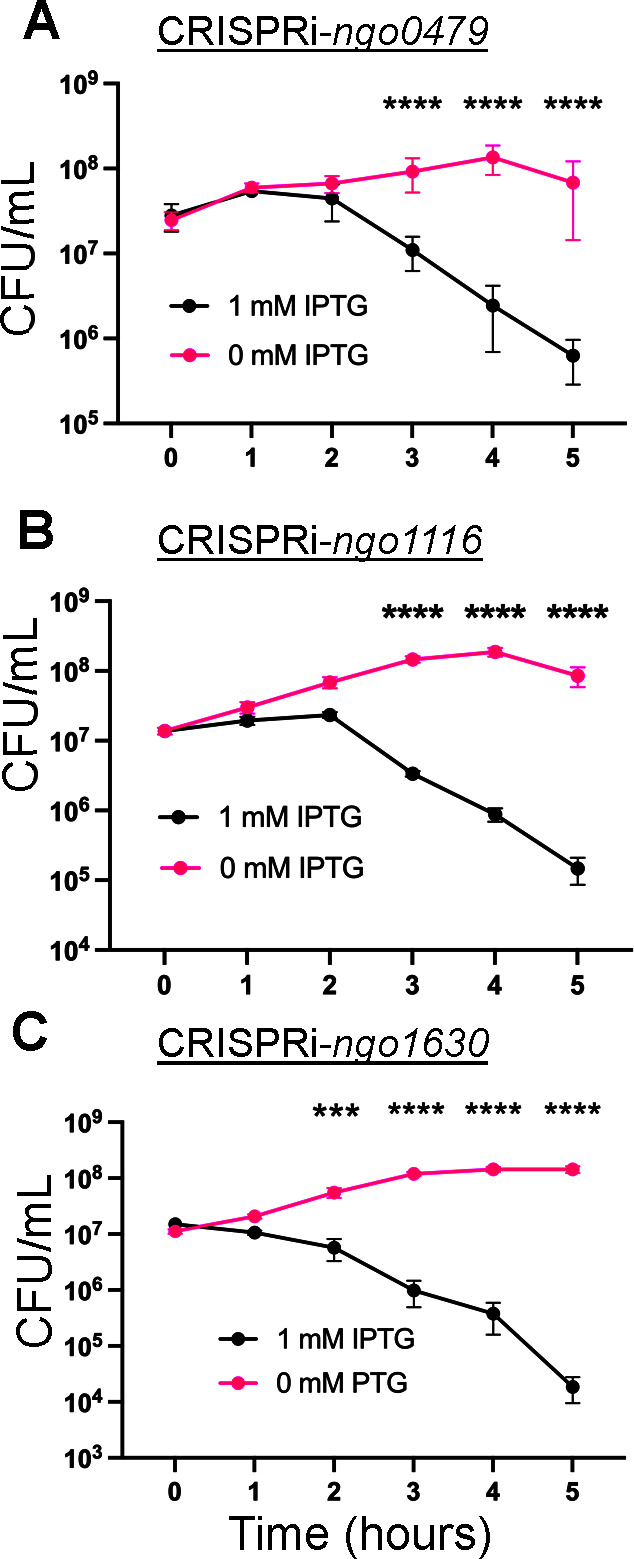
CRISPRi-mediated repression of *ngo0479*, *ngo1116*, or *ngo1630* is lethal. Gc survival assays using the Type I-C CRISPRi system to target (**A**) *ngo0479*, (**B**) *ngo1116*, and (**C**) *ngo1630*. Data are averages of three biological replicates, each with three technical replicates. Significance was determined using a two-way ordinary analysis of variation (panels A, B, and C: *P* < 0.0001, followed by Sídak’s multiple comparisons test). ****P < 0.0001 (panel A, 3–5 hours), ****P < 0.0001 (panel B, 3–5 hours), ***P = 0.0003 (panel C, 2 hours), ****P < 0.0001 (panel C, 3–5 hours).

Previous saturating transposon-insertional sequencing assays performed in two Gc isolates (FA1090 and MS11) did not target *ngo0479*, *ngo1116*, and *ngo1630*, suggesting that these genes are essential (21; Hu and Seifert, unpublished data). Since attempts to mutate these genes using insertional mutagenesis were unsuccessful, we conditionally repressed these genes using an isopropyl β-D-1-thiogalactopyranoside (IPTG)-inducible CRISPR-interference system (33). Without the induction of the CRISPRi machinery, strains carrying the CRISPR machinery targeting *ngo0479* (Fig. 3A), *ngo1116* (Fig. 3B), or *ngo1630* (Fig. 3C) grew normally during a 5 hour time course in liquid media. Upon adding 1.0 mM IPTG to these cultures, viability was unaffected for the first hour of growth but dropped significantly in all three strains after 2 hours of repression. These results show that the individual expression of *ngo0479*, *ngo1116*, or *ngo1630* is essential for Gc survival.

### Repression of the three *cI* orthologs leads to phage gene induction

**Fig 4:**
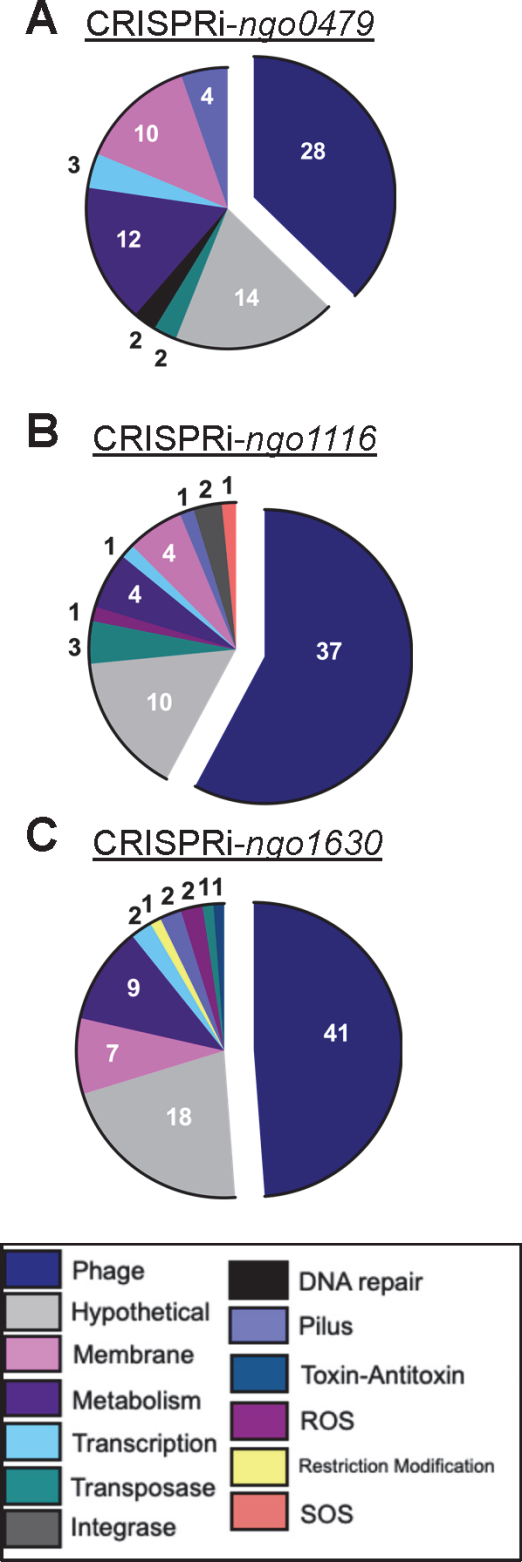
Knockdown of each *cI* ortholog leads to a significant change in prophage gene expression. The CRISPRi-*ngo0479* (**A**), CRISPRi-*ngo1116* (**B**), and CRISPRi-*ngo1630* (**C**) strains were grown in liquid gonococcal base medium (GCBL) media with or without 1.0 mM IPTG for 2 hours, followed by RNA isolation and RNA sequencing. The colors in the Venn diagrams denote the biological functional categories of genes displaying a significant difference in transcript abundance between the two growth conditions.

As *ngo0479*, *ngo1116*, and *ngo1630* are *cI* orthologs, to determine whether they regulate the expression of phage genes, we performed RNA sequencing with RNA isolated at the 2 hour growth timepoint after CRISPRi repression, when a reduction in viability first becomes apparent (Fig. 3). Knockdown of each *cI* ortholog results in a significant change in Gc prophage gene expression: 28 genes from CRISPRi-*ngo0479* (Fig. 4A), 37 genes from CRISPRi-*ngo1116* (Fig. 4B), and 41 genes from CRISPRi-*ngo1630* (Fig. 4C). Hypothetical genes, which we designated as genes with no predicted function that were encoded outside of the phage islands, were also differentially expressed upon the knockdown of *ngo0479* (14 genes), *ngo1116* (10 genes), and *ngo1630* (18 genes). Other functional groups with significant changes in gene expression included membrane-associated genes (*ngo0479*: 10 genes, *ngo1116*: 4 genes, *ngo1630*: 7 genes) and metabolism (*ngo0479*: 12, *ngo1116*: 4, *ngo1630*: 9). Genes involved in transcription, DNA repair, ROS survival, piliation, restriction-modification, as well as transposases, integrases, toxin-antitoxin genes, and Gc’s minimal SOS regulon, were also differentially expressed upon repression of each *cI* ortholog, albeit at lower numbers from 1 to 4 genes per category (39). We could not determine which of these transcriptional responses were due to the direct action of the repressor and which were induced due to lethality. Table S3A through C lists all the genes that display a significant difference in gene expression following the growth of each strain with versus without IPTG supplementation.

Almost all the phage genes that are differentially expressed are encoded in Ngoɸ1, Ngoɸ2, and Ngoɸ3 (Table S2). Furthermore, of the phage genes that are differentially expressed, most are induced: 86% following *ngo0479* repression, 89% following *ngo1116* repression, and 95% following *ngo1630* repression (Table S4). Many genes that differed in gene expression are paralogous to genes found in other phage islands, making it difficult to distinguish between genes.

By analyzing reads specific for each gene found in each island, we determined that repression of each *cI* ortholog results in the most phage gene changes corresponding to its respective island. For Ngoɸ1, *ngo0479* knockdown results in the differential gene expression of 14 genes—with 13 prophage genes induced and only *ngo0479* repressed. Knockdown of *ngo1116* and *ngo1630* results in 9 and 7 Ngoɸ1 genes, respectively, changing expression. For Ngoɸ2, CRISPRi-mediated *ngo1116* repression resulted in 19 genes (including *ngo1116*) changing expression, while 11 genes changed expression following *ngo0479* repression and 15 following *ngo1630* repression. This pattern, in which repressor knockdown results in the greatest expression change in genes encoded in each *cI*’s respective phage island, was repeated for Ngoɸ3, where *ngo1630* knockdown resulted in 18 genes being induced and only *ngo1630* being repressed. In comparison, only three genes in Ngoɸ3 changed expression following *ngo0479* knockdown, and nine genes in Ngoɸ3 changed expression after the *ngo1116* knockdown. These results suggest that each CI protein maintains the most control—either through direct or indirect regulation—over the other genes encoded in its local phage island. However, this analysis also shows that these repressors may cross-regulate the other island’s genes.

### Repression of *ngo0479*, *ngo1116*, and *ngo1630* leads to phage production

**Fig 5:**
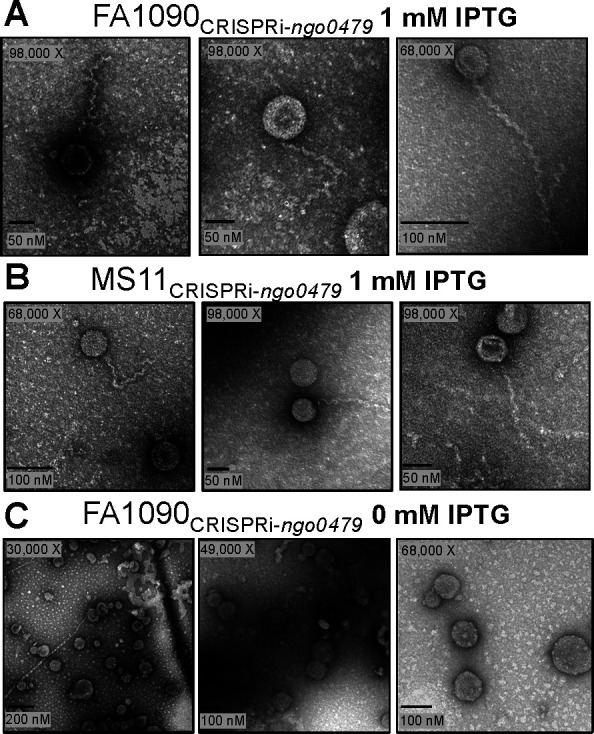
Phage particles are detected only upon *ngo0479* knockdown. Transmission electron microscopy images of phage particles isolated following overnight CRISPRi-mediated *ngo0479* repression from (**A**) FA1090 N-1-60 and (**B**) MS11 H-L-122. (**C**) TEM images of particles isolated from FA1090 CRISPRi-*ngo0479* grown overnight without IPTG treatment. The upper left value indicates the microscope magnification, and the bottom left is a scale bar with a reference measurement.

As the knockdown of the three *cI* genes leads to the induction of a significant number of phage genes in Ngoɸ1–3, we tested whether repressor knockdown was sufficient to produce phage. We prepared phage preparations from the CRISPRi-*ngo0479*, CRISPRi-*ngo1116*, and CRISPRi*-ngo1630* strains grown overnight in media supplemented with or without 1.0 mM IPTG. We used transmission electron microscopy (TEM) to visualize the negatively stained phage preps and found that each repressor’s knockdown produced phage particles with shared morphology (Fig. 5A; Fig. S3A and C). These TEM-imaged phage particles display characteristics consistent with the *Siphoviridae* family, defined by icosahedral heads and long, flexible tails (40). The capsid heads measured 71 nM (95% confidence interval [CI]: [68, 74]) in diameter, and the spiral tails were about 151 nM (95% CI: [143, 160]) in length—in agreement with known *Siphoviridae* size ranges (41–44). We also transferred the Type I-C CRISPRi system carrying the *ngo0479*-targeting spacer to the MS11 Gc isolate, which Phaster predicted as also encoding three similar dsDNA prophage islands. When the MS11 CRISPRi-*ngo0479* strain was repressed with IPTG, we observed phage particles with the same morphology as from FA1090 (Fig. 5B).

**Fig 6:**
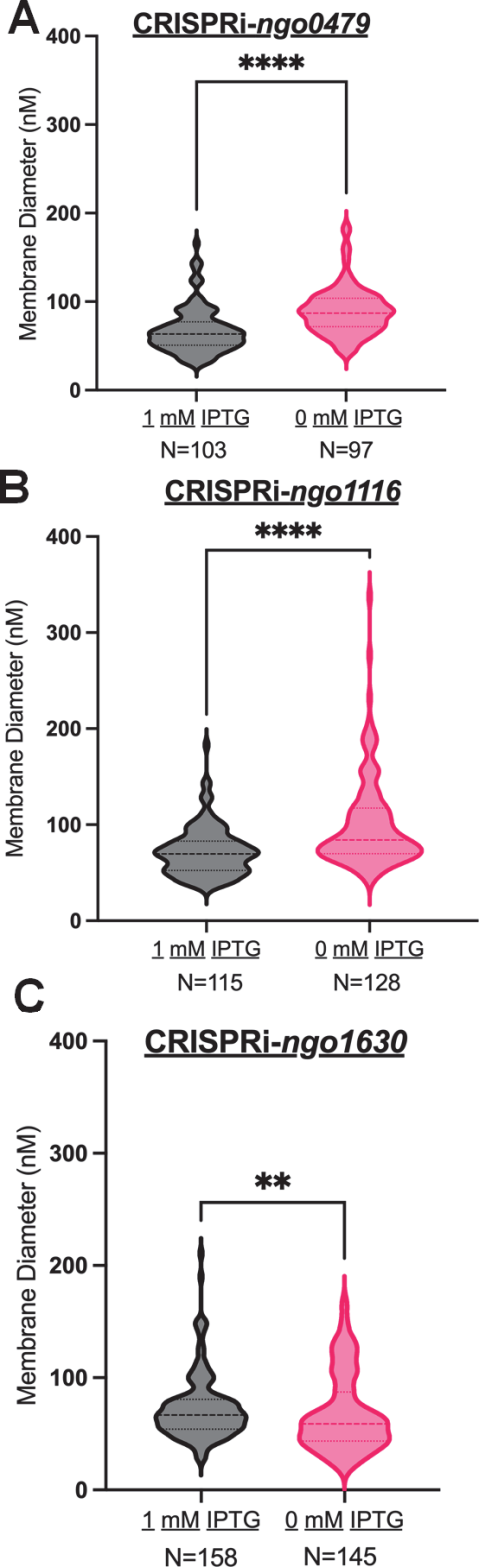
Repression of *ngo0479*, *ngo1116*, and *ngo1630* induces different-sized structures than particles released without *cI* repression. Violin plot comparing the distribution in diameter sizes of particles released from (**A**) CRISPRi-*ngo0479*, (**B**) CRISPRi-*ngo1116*, or (**C**) CRISPRi-*ngo1630* strains grown overnight in media supplemented with or without 1 mM IPTG. The number under each violin plot indicates the number of particles measured. Statistical significance was determined using the Mann-Whitney test comparing the mean particle diameter released from each strain with or without 1 mM IPTG treatment. ****P < 0.0001 (panels A and B), **P = 0.0068 (panel C). Within each violin plot, the boldest dashed line in the center represents the median, and the top and bottom dashed lines represent the 25th and 75th percentiles, respectively.

To determine whether these phages were produced due to repression of the *cI* orthologs—and not from the phage isolation protocol—we visualized preps from each CRISPRi strain grown without IPTG supplementation (Fig. 5C, Fig. 6; Fig. S2B and D). Absent from the images are the spiral tail structures. However, we detected larger amorphous membranous structures in the TEM images. We measured these structures and determined that the mean particle diameter following *cI* repression was 67.5 nM (*ngo0479*) (Fig. 6A), 71.9 nM (*ngo1116*) (Fig. 6B), and 72.7 nM (*ngo1630*) (Fig. 6C). On average, larger particles were isolated from the unrepressed CRISPRi-*ngo0479* (89.2 nM) and CRISPRi-*ngo1116* (99.3 nM), but smaller from CRISPRi-*ngo1630* (68.1 nM). The structures visualized from the non-repressed samples are less uniform than the phage capsids isolated after the repression of the three *cI* orthologs. These structures are most likely outer membrane vesicles (OMVs). *Neisseria* OMVs range from 20 to 200 nM, varying in size and shape depending on the isolation method (45–47). These results suggest that repression of *ngo0479*, *ngo1116*, and *ngo1630* leads to phage production.

## DISCUSSION

We show that the three *cI* orthologs (*ngo0479*, *ngo1116*, and *ngo1630*) encoded in Gc’s most complete dsDNA prophage islands (Ngoɸ1, Ngoɸ2, and Ngoɸ3) are essential and control phage production. We employed RNA sequencing to examine the transcriptional changes that occurred upon *ngo0479*, *ngo1116*, and *ngo1630* and found that most of the genes that changed expression were prophage genes from Ngoɸ1, Ngoɸ2, and Ngoɸ3. Furthermore, of the prophage genes that did change expression, the vast majority increased in expression upon *cI* repression. Finally, CRISPRi-mediated repression of *ngo0479*, *ngo1116*, and *ngo1630* each resulted in the production of *Siphoviridae*-like phage particles visible through TEM. Importantly, we show that only *cI* knockdown resulted in virion induction, with phage production not being an artifact of our isolation protocol. Thus, we hypothesize that the three *cI* orthologs are essential because they regulate and inhibit phage production in Gc.

This work represents the first demonstration of CI-controlled phage production in Gc. A previous study reported the presence of Lambda-like phage released from Gc (15). In this study, TEM was used to image a single phage particle released from Mitomycin C (MMC)-treated FA1090; however, only one image was included, and untreated bacteria were not visualized. This particle could be an OMV or an artifact from the phage isolation protocol. Thus, the lack of proper controls renders this study’s conclusions questionable. This study also investigated the ability of NGO0479 and NGO1116 to repress lytic expression of the *Escherichia coli*-encoded λ_cI*ts* 857_ phage. While the authors report that both CI orthologs reduce plaque formation, the ectopic expression of these genes negatively affected bacterial fitness. However, the reduction in host viability could be responsible for the decrease in plaques—not the result of NGO0479 or NGO1116 repressing lysis.

NGO0479, NGO1116, and NGO1630 are weak paralogs with overlapping but distinct regulons. Despite this, repression of each *cI* ortholog resulted in phage particles with similar morphologies. We could not isolate the phage DNA without contaminating genomic DNA and could not determine whether the phage isolated following repression of each *cI* differed in genetic composition. However, the knockdown of the three *cI* orthologs did lead to the highest induction of phage genes within each repressor’s respective island. This result suggests that we may have identified three unique but related phage species. Alternatively, components of each island may cooperate to produce a single phage. For example, the P4 *E. coli*-infecting phage can exist in the host alone as a plasmid but requires the structural gene products of the P2 helper phage to cause lytic infection (48, 49). The RNA-sequencing data do suggest island crosstalk, as repression of each *cI*—irrespective of which island the *cI* was encoded—resulted in changes in the gene expression from genes located in the other two islands. Interestingly, the CI orthologs did not seem to exhibit transcriptional control over each other, as knockdown in one *cI* did not affect the transcript of the other *cI* genes. Thus, this inter-island regulation may be caused by each regulator directly repressing genes within the other islands. Whether deleting any of these islands still allows phage production should be investigated.

What conditions trigger Gc phage production *in vivo*, or whether these islands remain dormant, is not clear. Temperature fluctuations (50–52), UV irradiation (53), and MMC (54) are frequently used to induce phage production in many bacterial species. Gc is restricted to the human body within narrow temperature ranges, so temperature fluctuation would be an unlikely condition to induce phage (55). UV irradiation is not a likely trigger for phage induction as Gc does not encounter it at mucosal surfaces. Despite encoding a *phrB* ortholog that, in other bacteria, functions as a photolyase for UV-induced damage repair, Gc’s *phrB* does not impact bacterial survival to UV irradiation (56). Indeed, Gc’s PhrB was likely co-opted for other functions because the bacteria rarely encounter UV. Finally, it is unlikely that Gc will ever naturally interact with MMC. Furthermore, we failed to induce phage production with Gc MMC treatment. More likely, hydrogen peroxide may induce Gc phage production under physiological conditions. Hydrogen peroxide is sufficient to induce phage production in many host species. Gc frequently encounters hydrogen peroxide released from neutrophils, and sublethal levels increase the expression of prophage genes (57). However, like MMC, we could not induce phage by treating Gc with H_2_O_2_. This failure to induce phage may be due to the H_2_O_2_ concentration we used or the amount of time we exposed the bacteria to the H_2_O_2_. Alternatively, there may be no natural condition that triggers Gc phage induction, and these islands may remain lysogens.

For this study, we limited our experimental manipulation to the Gc isolates FA1090 and MS11; however, other Gc isolates also encode similar islands. Through Phaster analysis, we determined that the Gc isolates FA19 (58) and NCCP11945 (59) also encode five predicted dsDNA prophages distributed throughout the chromosome. These isolates each encode about one or two predicted intact, degraded, and incomplete islands. Other Gc clinical isolates may also encode similar, inducible phage. Using PubMLST’s curated collection of clinical isolates, we found that of the over 32,000 Gc-characterized clinical isolates, 74% (24,097/32,762) retain an annotated copy of *ngo0479,* while 26% (8,665/32,762) do not (60). These data suggest that most sequenced clinical isolates may have inducible phage. To determine the conservation of these islands in other *Neisseria* species, we also used Phaster to predict prophage in *N. meningitidis* isolates MC58 (61), 8013 (62), Z2941 (63), and FAM18 (64). Interestingly, these *N. meningitidis* isolates each encode a single, complete island and one (8013, Z2941), two (FAM18), or no (MC58) degraded islands. Furthermore, all four isolates encode Mu-like phage, a *Myoviridae* species not found in the Gc isolates analyzed (65, 66). Thus, the Gc prophage appears to be dissimilar to those encoded in other *Neisseria*.

It would also be interesting to identify other bacteria permissive to Gc’s phage. Phage typically infects bacteria of the same or a closely related species due to the presence of conserved receptor-binding proteins (67), so it is more likely that the Gc phage from this study will infect a different *Neisseria* species or Gc isolate (68–70). We attempted both plaquing and transduction assays using the CRISPRi-*ngo0479* strain as the phage donor strain and Gc isolate 1291 (71)—which encodes dissimilar prophage islands to FA1090—as the receptor strain. While we did not detect lytic or lysogenic infection of 1291 in our assays, this result does not exclude the possibility that Ngoɸ1–3 may infect other Gc or *Neisseria* species.

We speculate that Gc may obtain fitness benefits from carrying these prophage islands and requires tight regulation by NGO0479, NGO1116, and NGO1630 to prevent lytic phage induction. Like many phage genomes, Ngoɸ1–3 encodes many small genes with no predicted function (72). Yet some of these undefined genes may provide important advantages to the bacteria. Shiga and diphtheria toxin genes were originally acquired from phage and have enhanced bacterial virulence (73, 74). In addition to utilizing phage to obtain and spread antibiotic resistance cassettes, prophage genes have also been shown to increase host resistance to various antimicrobials and adverse conditions (75–77). Gc is likely utilizing these prophage genes for an undiscovered purpose, with CI inactivation or degradation only occurring during specific environmental stimuli. Future studies should focus on determining the functions of these prophage genes and the conditions that induce NGO0479, NGO1116, and NGO1630 derepression.

## MATERIALS AND METHODS

### Bacterial strains and growth conditions

All strains (Table S1) used for this study were FA1090 (N-1-60) or MS11 (H-L-122) Gc isolate derivatives. The N-1-60 strain background carries *pilE* G4-lock and *pilC2* phase-locked mutations that allow constitutive expression of the 1-81-S2 *pilE* variant (78). The H-L-122 strain also carries the *pilE* G4-lock mutation and *pilC2*-phase lock. Additionally, all strains used for this study encoded the *Neisseria lactamica* (Nla) Type I-C CRISPR-interference system (33). Strains were grown in 37°C at 5% CO_2_ on Gonococcal (Gc) medium base (BD Difco) (36.25 g/L), agar (1.25 g/L), Kellogg Supplement I (22.2 mM glucose, 0.68 mM glutamine, 0.45 mM cocarboxylase), and Kellogg Supplement II (1.23 µM Fe(NO_3_)_3_) (79). For liquid growth, bacteria were grown at 37°C in GCBL media consisting of Bacto Proteose Peptone 3 (15 g/L), K_2_HPO_4_ (23 mM), KHPO_4_ (7.35 mM), NaCl (17.11 mM), Kellogg Supplements I and II, and Na. The GCBL was supplemented with Kellogg Supplements and NaCO_3_ (5 mM).

### Prophage island prediction and bioinformatic analysis

Gc’s dsDNA prophage island predictions were performed using Phaster (PHAge Search Tool Enhanced Release) (34) and a previous *in silico* analysis by Piekarowicz et al. (15). The Phaster program was used to analyze the FA1090 genome sequence (NC_002946.2), and phage island loci were identified using the results of this program. Genomic locations of filamentous phage islands NGOɸ6–9 were previously determined (15, 16, 18). Protein functional predictions for each prophage gene were determined using a combination of Phaster and the NCBI Conserved Domain (80) and InterPro Protein Sequence Classification (81) databases. Multiple sequence alignment comparing NGO0479, NGO1116, and NGO1630 was performed using the Clustal-Omega 1.2.4 program via UniProt (38).

### Construction of CRISPRi knockdown strains

CRISPRi strains were generated as previously described (33). Briefly, genes were scanned for 35 base pair targeting spacer sequences downstream of the consensus Nla Type I-C CRISPR PAM sites (5′TTT′3 or 5′TTC′3) were identified (Table S2). Spacer sequences were within 50 base pairs of the start codon and designed to bind the coding strand. To reprogram the spacer sequence, we utilized the Spacer Switch Plasmid, a vector encoding ~1,050 base pairs of homology to the CRISPRi locus in the Gc chromosome. Flanked between these homologous regions is the CRISPR array with the new targeting spacer linked to Cat 2-9 chloramphenicol resistance cassette (82). We used SOEing (Splice by Overhang Extension) PCR to change the targeting spacer of the Spacer Switch Plasmid (83). Primers were designed using NEBase Changer (Table S2), and the Q5 High-Fidelity polymerase (NEB) was used to generate the new Spacer Switch Plasmid followed by KDL treatment (T4 Kinase, DpnI, T4 Ligase) to facilitate ligation of the PCR product and degradation of the template plasmid. The new Spacer Switch Plasmids containing either the *ngo0479*, *ngo1116*, or *ngo1630* targeting spacers were then transformed into Gc and selected on chloramphenicol (1 µg/mL). Sequence of the Nla Type I-C CRISPRi Delivery Vector and the Spacer Switch Plasmid are available at Addgene.

### Growth assays

All survival assays were performed in liquid media. Gc was grown overnight on solid GCB plates for 19 hours at 37°C in 5% CO_2_ and suspended in GCBL supplemented with Kellogg Supplements I/II and 0.5 M NaCO_3_. Bacteria were grown for 2 hours at 37°C on a rotating incubator and then diluted with fresh GCBL. This process was repeated before being diluted back to an optical density 550 (OD_550_) of 0.1 and treated with or without 1 mM IPTG (Sigma). Bacteria were then grown at 37°C on a shaking incubator at 220 rpm. Samples were taken and diluted for CFU/mL calculations at 1 hour intervals.

### RNA isolation and sequencing

RNA was isolated from Gc grown in liquid media as previously described (33). Starting at an OD_550_ of 0.1, bacteria were grown for 2 hours with or without IPTG, treated with Bacteria RNA Protect (Qiagen), and then pelleted and frozen at −80°C. RNA isolation was performed with the RNeasy Mini Kit (Qiagen), and the RNA was treated with DNase I (Promega) to degrade genomic DNA. Illumina RNA sequencing and the intermediate RNA analysis were performed by the SeqCenter in Pittsburgh, PA. To differentiate between prophage paralogs that display changes in gene transcript abundance, the raw Illumina reads were analyzed using the Integrative Genomics Viewer software (84). If the change in reads was detected in a region unique to a particular paralog, it could then be determined that the change in transcript occurred in that gene and not in its paralogs. If transcript changes occurred in gene sequences shared by multiple paralogs, it was impossible to pinpoint which paralog changed expression.

### Phage particle isolation

The method for phage isolation was adapted from a previous protocol (17). Bacteria were grown overnight on GCB plates and subsequently suspended in GCBL supplemented with Kellogg I/II and NaCO_3_. Gc was grown for 2 hours before being diluted with fresh liquid media. This growth process was repeated three times before bacteria were diluted to an OD_550_ of 0.05 and grown overnight with or without 1 mM IPTG. For MMC and H_2_O_2_ treatments, N-1-60 were treated with 26 ng/mL MMC or 15 mM H_2_O_2_ and grown overnight. The next day, cultures were centrifuged at 4,500 rpm for 40 minutes at room temperature, and the supernatant was collected and filtered through a 0.45 µm PTFE filter (Fisher Scientific). The supernatant was treated with DNase I (Sigma) and RNase A (Sigma) for 3 hours on a rocking incubator at room temperature and then precipitated overnight at 4°C with 2.5 M NaCl with 20% PEG8000 solution (Teknova). The next day, the culture was centrifuged at 10,000 rpm for 1 hour at 4°C, and the pellet was resuspended in 1× phosphate buffered saline (PBS).

### Transmission electron microscopy

The phage suspension was spotted onto Formvar/Carbon 200 mesh copper grids (Electron Microscopy Sciences) and negatively stained with 2% uranyl acetate. The grid was visualized with the FEI Technai Spirit G2 120 kV transmission electron microscope. Image J software was used to measure the size of the phage particle heads, tails, and Gc outer membrane vesicles (85). To determine the average size of the phage isolated following *cI* gene repression, 376 total heads and 175 tails were counted.

### Statistics

Statistical analyses were performed using GraphPad Prism, and data were considered significant with *P* < 0.05. For liquid growth assays, three biological replicates were performed with results depicted as the means ± the standard error. Two-way analysis of variance followed by Sídak’s multiple comparison test was used to determine significance, as indicated in the figure legend. For RNA-sequencing experiments, significantly differentiated genes were identified as having a |logFC| > 1 and a *P* value of <0.05. Mann-Whitney tests were used to determine significance in comparison of the mean diameter size of membranes released from the CRISPRi-*ngo0479*, CRISPRi-*ngo1116*, and CRISPRi-*ngo1630* strains treated with and without 1 mM IPTG.

## SUPPLEMENTAL MATERIAL

Tables S1 to S3; Figures S1 and S2 jb.00049-25-s0001.pdf Strains, oligonucleotide sequences, phage gene analysis, gene overlap, and electron micrographs.

ASM does not own the copyrights to Supplemental Material that may be linked to, or accessed through, an article. The authors have granted ASM a non-exclusive, world-wide license to publish the Supplemental Material files. Please contact the corresponding author directly for reuse.
