# Supplementary material for: CRISPRi-mediated repression of three cI repressors induces the expression of three related Neisseria gonorrhoeae bacteriophages
Source: J Bacteriol. 2025 May 12;207(6):e00049-25. doi: 10.1128/jb.00049-25 (PMC12186493; doi:10.1128/jb.00049-25)
Supplement: Tables S1 to S3; Figures S1 and S2 — Strains, oligonucleotide sequences, phage gene analysis, gene overlap, and electron micrographs. [file jb.00049-25-s0001.pdf]

1

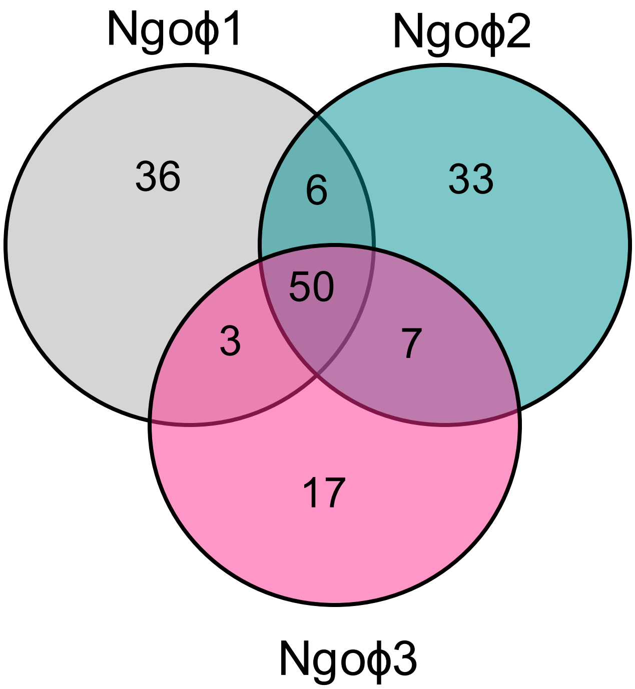

Supplemental Figure 1. NGOϕ1, NGOϕ2, and NGOϕ3 share a significant number of paralogous genes. Venn Diagram comparing the number of unique genes within each Gc phage island and the paralogous genes shared between the three islands.

2A FA1090$_{\text{CRISPRi-}\textit{ngo1116}}$ **1 mM IPTG**

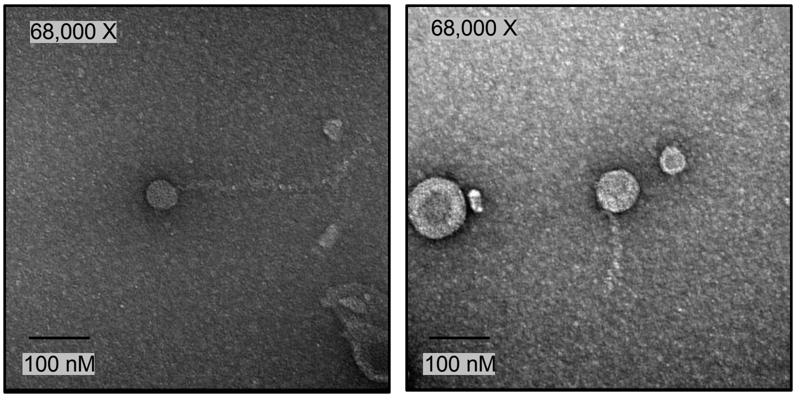

2B FA1090$_{\text{CRISPRi-}\textit{ngo1116}}$ **0 mM IPTG**

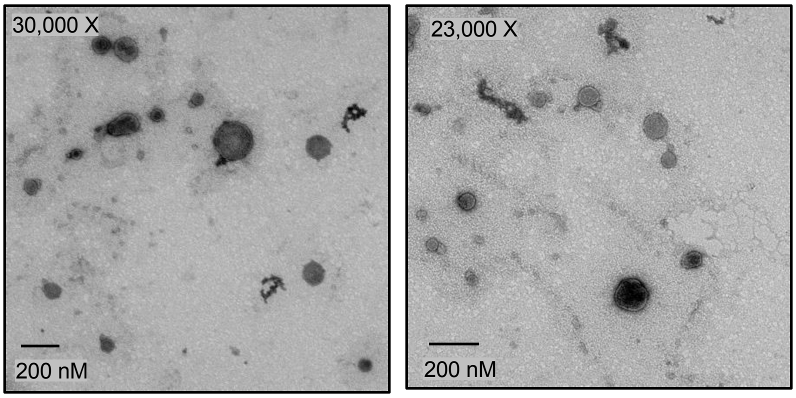

2C FA1090$_{\text{CRISPRi-}\textit{ngo1630}}$ **1 mM IPTG**

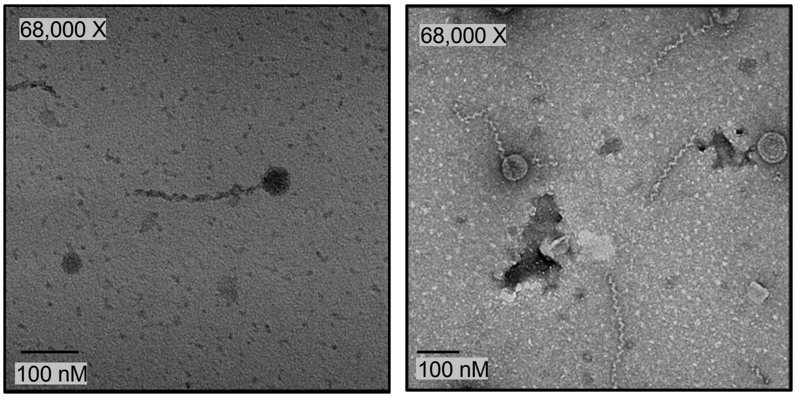

2D FA1090$_{\text{CRISPRi-}\textit{ngo1630}}$ **0 mM IPTG**

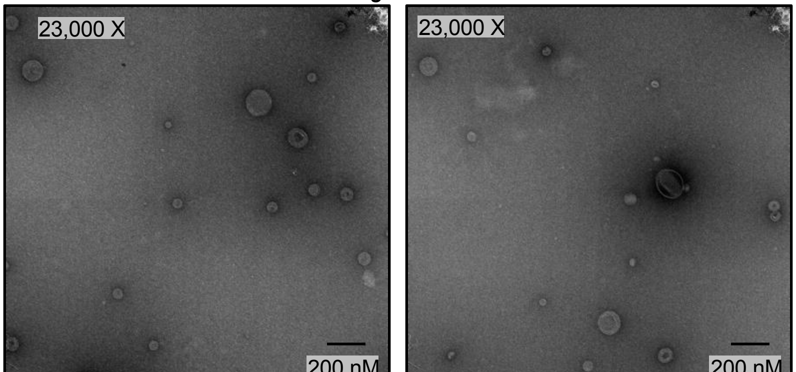

Supplemental Figure 2. Phage particles are detected only upon ngo1116 and ngo1630 knockdown. Transmission Electron Microscopy images of phage particles isolated following overnight CRISPRi-mediated *ngo1116* (A) and *ngo1630* (C) repression in the N-1-60 background. TEM images of particles isolated from N-1-60 CRISPRi-*ngo1116* (B) and N-1-60 CRISPRi-*ngo1630* (D) grown overnight without IPTG treatment. The upper left value indicates the magnification, and the bottom left is a scale bar with a reference measurement.

Supplemental Table 1. Strains, spacer sequences, and oligonucleoties used in this study.

Strain Name	Species	Strain	Description	Source
N-1-60	*N. gonorrhoeae*	FA1090	G4 multisite mutation, PilC2 phase-locked on	64
H-1-22	*N. gonorrhoeae*	MS11	G4 multisite mutation, PilC2 phase-locked on	Dr. Jayaram Narayana
CRISPRi-*ngo0479*	*N. gonorrhoeae*	FA1090	N-1-60 background with Type I-C CRISPRi gene loci, *ngo0479* targeting spacer	This paper
CRISPRi-*ngo1116*	*N. gonorrhoeae*	FA1090	N-1-60 background with Type I-C CRISPRi gene loci, *ngo1116* targeting spacer	This paper
CRISPRi-*ngo1630*	*N. gonorrhoeae*	FA1090	N-1-60 background with Type I-C CRISPRi gene loci, *ngo1630* targeting spacer	This paper
CRISPR-*ngo0479* (H-1-22)	*N. gonorrhoeae*	MS11	with Type I-C CRISPRi gene loci, *ngo0479* targeting spacer	This paper

Spacer Name	Sequence (5' to 3')	PAM sequence
ngo0479	gatctccgaattaattgcgcataagcattggtaat	TTT
ngo1116	aaattcagacatatcaaactccttataacgcaacg	TTT
ngo1630	agccgtctcaatttcatttaattctcttttttca	TTC

Oligonucleotide	Sequence (5' to 3')	Description	Source
NGO0479_Spacer_F1	caattaattcggagatcGTTTCAACACACAGCCGC	Oligonucleotide containing part of *ngo0479* spacer and CRISPRi repeat	This study
NGO0479_Spacer_R1	cgcataagcattggtaatTTTTTTTTCTAGATATCGCTCAATACTG	Oligonucleotide containing part of *ngo0479* spacer and CRISPRi repeat	This study
NGO1116_Spacer_F1	ttgatatgtctgaatttGTTTCAACACACAGCCGC	Oligonucleotide containing part of *ngo1116* spacer and CRISPRi repeat	This study
NGO1116_Spacer_R1	actccttataacgcaacgTTTTTTTTCTAGATATCGCTCAATACTG	Oligonucleotide containing part of *ngo1116* spacer and CRISPRi repeat	This study
NGO1630_Spacer_F1	atgaaattgagacggctGTTTCAACACACAGCCGC	Oligonucleotide containing part of *ngo1630* spacer and CRISPRi repeat	This study
NGO1630_Spacer_R1	ttaattctcttttttcaTTTTTTTTCTAGATATCGCTCAATACTG	Oligonucleotide containing part of *ngo1630* spacer and CRISPRi repeat	This study

Supplemental Table 2. List of genes encoded in NGOϕ1, NGOϕ2, and NGOϕ3. Genes were listed according to prophage island, predicted function, whether the gene is unique or paralogous to another phage gene, the degree of similarity between paralogs, and what islands the paralogs are encoded. The genes comprising NGOϕ6 and NGOϕ9 are noted.

Phage Island	Gene	Predicted Function	Unique or Paralog?	% Identity/%coverage to paralog?	Phage Island Designation of Paralog	Note
1	NGO0459		Unique			
1	NGO0460		Unique			
1	NGO0461		Unique			
1	NGO0462	Integrase	Unique			
1	NGO0463		Unique			
1	NGO0464		Unique			
1	NGO0465		Paralog	NGO1616 90%/49%<br>NGO1130 90%/27%<br>NGO1615 82%/17%	2, 3	
1	NGO0466		Paralog	NGO1617 100%/99%<br>NGO1129 100%/99%	2, 3	
1	NGO0467		Paralog	NGO1618 98%/100%<br>NGO1128 98%/100%	2, 3	
1	NGO0468		Paralog	NGO1619 98%/100%<br>NGO1127 98%/100%	2, 3	
1	NGO0469	Replication Initiation	Paralog	NGO1620 100%/100%<br>NGO1126 100%/82%	2,3	
1	NGO0470		Paralog	NGO1621 100%/100%<br>NGO1125 100%/100%	2, 3	
1	NGO0471		Paralog	NGO1622 100%/100%<br>NGO1125 100%/100%	2, 3	
1	NGO0472		Paralog	NGO1623 96%/ 100%<br>NGO1123 96%/ 100%	2, 3	
1	NGO0473		Paralog	NGO1122 100%/100%	2	
1	NGO0474		Paralog	NGO1121 100%/100%	2	
1	NGO0475		Paralog	NGO1624 88%/ 100%<br>NGO1120 98%/ 100%	2, 3	
1	NGO0476		Unique			
1	NGO0477	Transcriptional repressor	Unique			
1	NGO0478		Unique			
1	NGO0479	Transcriptional Repressor	Unique			
1	NGO0479.1		Unique			
1	NGO0480		Paralog	NGO1631 100%/ 100%<br>NGO1114 100%/ 100%	2, 3	
1	NGO0481		Paralog	NGO1632 100%/ 100%<br>NGO1113 100%/ 100%	2, 3	
1	NGO0482		Paralog	NGO1632 85%/ 97%<br>NGO1112 100%/ 100%	2, 3	

Phage Island	Gene	Predicted Function	Unique or Paralog?	% Identity/%coverage to paralog?	Phage Island Designation of Paralog	Note
1	NGO0483		Paralog	NGO1111.1 100%/100%	2	
1	NGO0484	Phage DNA replication protein	Paralog	NGO1111 93%/100%	2	
1	NGO0485	DNA Helicase dnaB	Paralog	NGO1636 35%/7%<br>NGO1110 99%/100%	2, 3	
1	NGO0486		Paralog	NGO1109 100%/100%	2	
1	NGO0487		Paralog	NGO1638 100%/100%<br>NGO1108 100%/100%	2, 3	
1	NGO0488	Putative hollin	Paralog	NGO1639 96%/98%<br>NGO1106 96%/98%	2, 3	
1	NGO0489	Endodeoxyribon uclease RusA	Paralog	NGO1640 40%/27%<br>NGO1105 40%/27%<br>NGO0490 43%/25%	2, 3	
1	NGO0490	Endodeoxyribon uclease RusA	Paralog	NGO1640 94%/66%<br>NGO1105 94%/97%<br>NGO0489 43%/25%	2,3	
1	NGO0491		Unique			
1	NGO0492		Unique			
1	NGO0493		Unique			
1	NGO0494	Terminase small subunit	Unique			
1	NGO0495	Terminase large subunit	Unique			
1	NGO0496	Phage Portal Protein	Unique			
1	NGO0497	Protease Strucutal Protein	Unique			
1	NGO0498		Unique			
1	NGO0499		Unique			
1	NGO0500		Unique			
1	NGO0501		Unique			
1	NGO0502		Unique			
1	NGO0503		Unique			
1	NGO0504		Unique			
1	NGO0505		Unique			
1	NGO0506		Unique			
1	NGO0507		Unique			
1	NGO0508		Unique			
1	NGO0509	HTH transcriptional repressor	Unique			

Phage Island	Gene	Predicted Function	Unique or Paralog?	% Identity/%coverage to paralog?	Phage Island Designation of Paralog	Note
1	NGO0510	Phage Tail Protein	Unique			
1	NGO0511		Unique			
1	NGO0513		Unique			
1	NGO0514		Unique			
1	NGO0515		Unique			
1	NGO0516	Growth Inhibitor PemK	Unique			
1	NGO0517	PemI-like protein	Unique			
1	NGO0518	Endolysin	Paralog	NGO1649 83%/100%	3	
1	NGO0519		Paralog	NGO1650 100%/100%	3	
1	NGO0520		Paralog	NGO1651 100%/100%	3	
1	NGO0521		Paralog	NGO1086 64%/100%	2	
1	NGO0522	Tail length tape measure protein	Unique			
1	NGO0523		Unique			
1	NGO0524	Integrase	Unique			
1	NGO0526	phosphoribosylformylglycinamidine cyclo-ligase	Unique			
2	NGO1085	Antirepressor	Paralog	NGO1652 68%/99%	3	
2	NGO1086		Paralog	NGO0521 64%/100%	1	
2	NGO1087		Unique			
2	NGO1088		Unique			
2	NGO1089	Phage Structural Protein	Unique			
2	NGO1090	Phage Structural Protein	Unique			
2	NGO1091		Unique			
2	NGO1092	DNA Methylase	Unique			
2	NGO1093		Unique			
2	NGO1094		Unique			
2	NGO1095		Unique			
2	NGO1097	Phage Portal Protein	Unique			
2	NGO1098	Phage terminase large subunit	Unique			
2	NGO1099		Unique			
2	NGO1100	Terminase small subunit	Unique			
2	NGO1101		Unique			

Phage Island	Gene	Predicted Function	Unique or Paralog?	% Identity/%coverage to paralog?	Phage Island Designation of Paralog	Note
2	NGO1102	HNH Endonuclease family protein	Unique			
2	NGO1103	NinB	Unique			
2	NGO1104		Unique			
2	NGO1105	Endodeoxyribonuclease RusA	Paralog	NGO1640 97%/89%<br>NGO0489 40%/27%<br>NGO0490 43%/25%	1, 3	
2	NGO1106	Putative hollin	Paralog	NGO1639 100%/100%<br>NGO0488 96%/98%	1, 3	
2	NGO1108		Paralog	NGO1638 100%/100%<br>NGO0487 100%/100%	1, 3	
2	NGO1109		Paralog	NGO0486 100%/100%	1	
2	NGO1110		Paralog	NGO1636 35%/7%<br>NGO0485 99%/100%	1, 3	
2	NGO1111	Phage DNA replication protein	Paralog	NGO0484 93%/100%	1	
2	NGO1111.1		Paralog	NGO0483 100%/100%	1	
2	NGO01112		Paralog	NGO1633 85%/97%<br>NGO0482 100%/ 100%	1, 3	
2	NGO1113		Paralog	NGO1632 100%/100%<br>NGO0481 100%/ 100%	1, 3	
2	NGO1114		Paralog	NGO1631 100%/ 100%<br>NGO0480 100%/ 100%	1, 3	
2	NGO1115		Unique			
2	NGO1116	Transcriptional Repressor	Unique			
2	NGO1117		Unique			
2	NGO1118		Unique			
2	NGO1119		Unique			
2	NGO1120		Paralog	NGO1624 86%/100%<br>NGO0475 98%/100%	1, 3	
2	NGO1121		Paralog	NGO0474 100%/100%	1	
2	NGO1122		Paralog	NGO0473 100%/100%	1	

Phage Island	Gene	Predicted Function	Unique or Paralog?	% Identity/%coverage to paralog?	Phage Island Designation of Paralog	Note
2	NGO1123		Paralog	NGO1623 100%/100%<br>NGO0472 96%/100%	1, 3	
2	NGO1124		Paralog	NGO1622 100%/100%<br>NGO0471 100%/100%	1, 3	
2	NGO1125		Paralog	NGO1621 100%/100%<br>NGO0470 100%/100%	1,3	
2	NGO1126	Phage DNA replication initiation	Paralog	NGO1620 100%/80%<br>NGO0469 100%/80%	1,3	
2	NGO1127		Paralog	NGO1619 100%/100%<br>NGO0468 98%/100%	1,3	
2	NGO1128		Paralog	NGO1618 100%/100%<br>NGO0467 98%/100%	1,3	
2	NGO1129		Paralog	NGO1617 100%/100%<br>NGO0466 100%/100%	1,3	
2	NGO1130		Paralog	NGO1616 100%/100%<br>NGO0465 90%/27%	1, 3	
2	NGO1131		Paralog	NGO1615 93%/38%<br>NGO1616 64%/15%	3	
2	NGO1132		Unique			
2	NGO1133		Unique			
2	NGO1134		Unique			
2	NGO1135		Unique			
2	NGO1136		Unique			
2	NGO1137	Transposase	Paralog	NGO1641 100%/100%	3	NGOϕ6
2	NGO1137.1		Paralog	NGO1642 100%/100%	3	
2	NGO1138		Paralog	NGO1643 100%/100%	3	
2	NGO1139		Paralog	NGO1644 100%/100%	3	
2	NGO1140	TspB	Paralog	NGO1645 100%/52%	3	
2	NGO1141		Unique			
2	NGO1142		Unique			
2	NGO1143		Unique			
2	NGO1144		Unique			
2	NGO1145		Unique			
2	NGO1146	DNA relaxase NicK	Unique			

Phage Island	Gene	Predicted Function	Unique or Paralog?	% Identity/%coverage to paralog?	Phage Island Designation of Paralog	Note
3	NGO1610		Unique			
3	NGO1610.1		Unique			
3	NGO1610.2		Unique			
3	NGO1611		Unique			
3	NGO1612		Unique			
3	NGO1613	Integrase	Unique			
3	NGO1614		Unique			
3	NGO1615		Paralog	NGO1131 93%/7%<br>NGO0465 82%/17%	1, 2	
3	NGO1616		Paralog	NGO1130 100%/100%<br>NGO1131 63%/3%<br>NGO0465 90%/27%	1,2	
3	NGO1617		Paralog	NGO1129 100%/100%<br>NGO0466 100%/100%	1,2	
3	NGO1618		Paralog	NGO1128 100%/100%<br>NGO0467 98%/100%	1,2	
3	NGO1619		Paralog	NGO1127 100%/100%<br>NGO0468 98%/100%	1,2	
3	NGO1620	Replication Initiation	Paralog	NGO1126 100%/82%<br>NGO0469 100%/100%	1,2	
3	NGO1621		Paralog	NGO1125 100%/100%<br>NGO0470 100%/100%	1,2	
3	NGO1622		Paralog	NGO1124 100%/100%<br>NGO0471 100%/100%	1,2	
3	NGO1623		Paralog	NGO1123 100%/100%<br>NGO0472 96%/100%	1,2	
3	NGO1624		Paralog	NGO1120 87%/100%<br>NGO0475 88%/100%	1,2	
3	NGO1626		Unique			
3	NGO1627		Unique			
3	NGO1628		Unique			
3	NGO1629		Unique			

Phage Island	Gene	Predicted Function	Unique or Paralog?	% Identity/%coverage to paralog?	Phage Island Designation of Paralog	Note
3	NGO1630	Transcriptional Repressor	Unique			
3	NGO1631		Paralog	NGO1114 100%/100%<br>NGO0480 100%/100%	1,2	
3	NGO1632		Paralog	NGO1113 94%/100%<br>NG00481 94%/100%	1,2	
3	NGO1633		Paralog	NGO1112 85%/97%<br>NGO0482 85%/97%	1,2	
3	NGO1634		Unique			
3	NGO1635		Unique			
3	NGO1636	Phage Replication Protein	Unique			
3	NGO1638		Paralog	NGO1108 100%/100%<br>NGO0487 100%/100%	1,2	
3	NGO1639	Putative hollin	Paralog	NGO1106 100%/100%<br>NGO0486 96%/100%	1,2	
3	NGO1640	Holliday junction resolvase RusA	Paralog	NGO1105 97%/98%<br>NGO0490 94%/66%	1,2	
3	NGO1641	Transposase	Paralog	NGO1137 100%/100%	2	NGOϕ9
3	NGO1642		Paralog	NGO1137.1 100%/100%	2	
3	NGO1643		Paralog	NGO1138 100%/100%	2	
3	NGO1644		Paralog	NGO1139 100%/100%	2	
3	NGO1645	TspB	Paralog	NGO1140 100%/100%	2	
3	NGO1646		Unique			
3	NGO1647		Paralog	NGO1643 93%/91%<br>NGO1138 93%/91%	2,3	
3	NGO1648	Transposase	Unique			
3	NGO1649	Endolysin	Paralog	NGO0518 83%/100%	1	
3	NGO1650		Paralog	NGO0519 100%/100%	1	
3	NGO1651		Paralog	NGO0520 100%/100%	1	
3	NGO1651.1	type II toxin-antitoxin system PemK/MazF family toxin	Unique			
3	NGO1652	Antirepressor	Paralog	NGO1085 68%/99%	2	

Supplemental Table 3. Genes displaying a difference in transcript abundance in the CRISPRi-*ngo0479*, CRISPRi-*ngo1116*, and CRISPRi-*ngo1630* strains following growth in 1.0 versus 0 mM IPTG. List of genes identified through RNA-sequencing that change transcript abundance following repression of each *cI* ortholog, according to locus tag, gene name, Log2Fc, p-value, and known or predicted function.

3A

CRISPRi-*ngo0479*; Change in gene transcript abundance (1 mM vs. 0 mM IPTG)				
**Locus/Locus Tag[1]**	**Gene Name**	**log2FC**	**p value**	**Functional Classification[2]**
NGO_0463	-	2.4624079	0.0011562	Phage
NGO_0464	-	1.8835962	0.0060225	Phage
NGO_0465	-	3.2369056	0.0000240	Phage
NGO_0467	-	2.2293231	0.0082341	Phage
NGO_0472	-	4.9431027	0.0000019	Phage
NGO_0474	-	3.4466753	0.0000113	Phage
NGO0479	-	-3.6033313	0.0000015	Phage
NGO0479.1	-	3.0587126	0.0035675	Phage
NGO_0485	-	1.9783451	0.0079368	Phage
NGO_0487	-	1.7484974	0.0378812	Phage
NGO_0489	-	1.9599884	0.0380057	Phage
NGO_0490	RusA	1.7172451	0.0298260	Phage
NGO_0503	-	1.8893501	0.0264292	Phage
NGO_0506	-	4.2703277	0.0030428	Phage
NGO_1093	-	3.4527295	0.0007471	Phage
NGO_1094	-	1.7043176	0.0490758	Phage
NGO_1105	RusA	3.4475790	0.0399705	Phage
NGO_1119	-	-2.7602068	0.0047504	Phage
NGO_1121	-	5.3290526	0.0000000	Phage
NGO_1124	-	-5.8159214	0.0347826	Phage
NGO_1126	-	2.9982504	0.0003126	Phage
NGO_1131	-	-3.9933612	0.0013343	Phage
NGO_1141	-	6.9578421	0.0014090	Phage
NGO_1143	-	6.2646375	0.0140787	Phage
NGO_1145	-	3.0670670	0.0222081	Phage
NGO_1262	-	2.8281674	0.0396963	Phage
NGO_1620	-	6.1702764	0.0000003	Phage
NGO_1645	-	2.8281674	0.0396963	Phage
NGO_03840	-	2.4003839	0.0295884	Phage
NGO_0432	-	2.5667200	0.0017234	Hypothetical
NGO_0896	-	1.4820847	0.0410925	Hypothetical
BCHEFKBB_01032	-	6.8433317	0.0019960	Hypothetical
NGO_1070	-	1.3867428	0.0453130	Hypothetical
NGO_1176	-	7.5752629	0.0001256	Hypothetical
BCHEFKBB_01298	-	3.0670670	0.0222081	Hypothetical
BCHEFKBB_01374	-	2.3026832	0.0196150	Hypothetical
NGO_1384	-	1.6721097	0.0287777	Hypothetical
NGO_1450	-	2.7645155	0.0000789	Hypothetical
BCHEFKBB_01494	-	1.8529046	0.0113933	Hypothetical
BCHEFKBB_01699	-	1.5596303	0.0243202	Hypothetical
BCHEFKBB_01922	-	2.4003839	0.0295884	Hypothetical

CRISPRi-*ngo0479;* Change in gene transcript abundance (1 mM vs. 0 mM IPTG)				
**Locus/Locus Tag[1]**	**Gene Name**	**log2FC**	**p value**	**Functional Classification[2]**
NGO_0463	-	2.4624079	0.0011562	Phage
NGO_0464	-	1.8835962	0.0060225	Phage
NGO_0465	-	3.2369056	0.0000240	Phage
NGO_0467	-	2.2293231	0.0082341	Phage
NGO_0472	-	4.9431027	0.0000019	Phage
NGO_0474	-	3.4466753	0.0000113	Phage
NGO0479	-	-3.6033313	0.0000015	Phage
NGO0479.1	-	3.0587126	0.0035675	Phage
NGO_0485	-	1.9783451	0.0079368	Phage
NGO_0487	-	1.7484974	0.0378812	Phage
NGO_0489	-	1.9599884	0.0380057	Phage
NGO_0490	RusA	1.7172451	0.0298260	Phage
NGO_0503	-	1.8893501	0.0264292	Phage
NGO_0506	-	4.2703277	0.0030428	Phage
NGO_1093	-	3.4527295	0.0007471	Phage
NGO_1094	-	1.7043176	0.0490758	Phage
NGO_1105	RusA	3.4475790	0.0399705	Phage
NGO_1119	-	-2.7602068	0.0047504	Phage
NGO_1323	ProQ	-1.8469948	0.0064890	Metabolism
NGO_0990	-	2.4120716	0.0005680	Transcription
NGO_0025	MpeR	2.2358966	0.0237107	Transcription
NGO_0262	GreB	-1.5330817	0.0311258	Transcription
NGO_04640	-	-1.7353628	0.0394505	Transposase
NGO_1394	-	-3.0348899	0.0015655	Transposae
NGO_07995	-	-1.6737700	0.0146942	Pilin
BCHEFKBB_01933	-	1.5178110	0.0245488	Pilin
NGO_11130	-	1.5803170	0.0455573	Pilin
NGO_11165	-	3.9729450	0.0074474	Pilin
NGO_0153	RuvC	-1.5098833	0.0307822	DNA repair
NGO_1000	Cas4	1.9453575	0.0228861	Exonuclease

1. If NGO number not assigned, locus tag assigned instead

2. Based on predicted or known functions

3B

CRISPRi-*ngo1116*; Change in gene transcript abundance (1 mM vs. 0 mM IPTG)				
Locus/Locus Tag[1]	Gene Name	log2FC	p value	Functional Classification[2]
NGO_0469	-	6.03184801	0.021900161	Phage
NGO_0474	-	4.81448478	5.11E-09	Phage
NGO_0484	-	2.33930353	0.023239034	Phage
NGO_0485	-	1.57534572	0.028335216	Phage
NGO_0486	-	6.53979099	0.006149306	Phage
NGO_0487	-	2.61713983	0.002786776	Phage
NGO_0497	-	-2.13241247	0.028123146	Phage
NGO_0498	-	2.19355715	0.01990983	Phage
NGO_0504	-	6.80023874	0.002863891	Phage
NGO_1007	-	1.64985951	0.044262584	Phage
NGO_1098	-	1.602786907	0.020673337	Phage
NGO_1101	-	1.510718406	0.040282674	Phage
NGO_1102	-	4.158161624	2.29E-05	Phage
NGO_1104	-	4.198661119	0.003042849	Phage
NGO_1105	RusA	3.901344592	0.007447428	Phage
NGO_1108	-	2.787431518	0.000281529	Phage
NGO_1109	-	6.914719852	0.001996045	Phage
NGO_1110	-	4.335383875	4.47E-08	Phage
NGO_1111	-	2.584259056	0.000554103	Phage
NGO_1114	-	2.041987001	0.049335813	Phage
NGO_1116	-	-4.637046037	1.53E-09	Phage
NGO_1121	-	4.525893832	3.32E-08	Phage
NGO_1128	-	2.23328937	0.005801967	Phage
NGO_1129	-	2.608972952	0.006974917	Phage
NGO_1130	-	1.403767008	0.049361659	Phage
NGO_1131	-	2.34964267	0.001105226	Phage
NGO_1132	-	3.684092723	2.53E-06	Phage
NGO_1137	-	2.487574009	0.000326976	Phage
NGO_1141	-	8.729238358	4.00E-07	Phage
NGO_1265	TspB	3.413538772	0.00025588	Phage
NGO_1610.1	-	-6.56236198	1.32E-12	Phage
NGO_1616	-	1.702997672	0.014085824	Phage
NGO_1618	-	4.403751738	6.56E-06	Phage
NGO_1619	-	2.440703167	0.022208097	Phage
NGO_1622	-	-6.743053024	0.002863891	Phage
NGO_1623	-	2.359263572	0.009435984	Phage
NGO_1624	-	-6.644479053	0.002863891	Phage
NGO_1638	-	1.490747079	0.046203229	Phage
NGO_0022	-	6.80023874	0.002863891	Hypothetical
NGO_0418	-	2.547855059	0.008474583	Hypothetical

CRISPRi-*ngo1116;* Change in gene transcript abundance (1 mM vs. 0 mM IPTG)				
BCHEFKBB_00977	-	-6.644479053	0.002863891	Hypothetical
NGO_1395	-	-2.137477183	0.014318505	Hypothetical
NGO_1450	-	3.49609231	1.34E-06	Hypothetical
BCHEFKBB_01699	-	1.66324708	0.020193325	Hypothetical
NGO_1700	-	-1.901950393	0.004883302	Hypothetical
NGO_09325	-	1.991468344	0.01580778	Hypothetical
NGO_1848	-	3.413538772	0.00025588	Hypothetical
BCHEFKBB_01918	-	4.451640949	4.35E-07	Hypothetical
BCHEFKBB_01922	-	7.64791689	8.54E-14	Hypothetical
NGO_1063	SliC	1.606823723	0.016592731	Membrane
NGO_1192	LrgA	2.23465977	0.001218571	Membrane
BCHEFKBB_01312	HmbR	1.437677076	0.034158909	Membrane
NGO_1359		2.246842678	0.029721761	Membrane
NGO_2137	MetN	-1.54058413	0.02176655	Membrane
BCHEFKBB_01162	-	6.539790994	0.006149306	Metabolism
NGO_1292	DsbB	1.858686156	0.005946776	Metabolism
BCHEFKBB_01407	-	-1.641303855	0.014821447	Metabolism
NGO_1936	-	-1.349851881	0.04229985	Metabolism
NGO_1980	-	1.474655508	0.027078719	Metabolism
NGO_0990	-	2.242383215	0.0011995	Transcription
NGO_1157	-	-1.807454859	0.029826037	Transposase
NGO_08295	-	4.522256668	4.13E-08	Transposase
NGO_1317	-	-1.793136662	0.022943106	Transposase
NGO_1769	CcpR	-1.31872565	0.04733234	ROS
NGO_1428	-	-1.474342368	0.036818783	SOS
NGO_10995	-	1.906679583	0.023091217	Pilus

1. If NGO number not assigned, locus tag assigned instead

2. Based on predicted or known functions

3C

CRISPRi-*ngo1630;* Change in gene transcript abundance (1 mM vs. 0 mM IPTG)				
Locus/Locus Tag[1]	Gene Name	log2FC	p value	Functional Classification[2]
NGO_0465	-	2.78114223	0.000633856	Phage
NGO_0467	-	2.987547045	0.018258403	Phage
NGO_0468	-	2.85097141	0.023239034	Phage
NGO_0469	-	9.96258581	2.59E-10	Phage
NGO_0470	-	6.731919567	0.002863891	Phage
NGO_0472	-	2.264412533	0.028743676	Phage
NGO_0487	-	1.965202672	0.014453768	Phage
NGO_1004	-	1.685299383	0.030442387	Phage
NGO_1105	RusA	3.809466019	0.000814859	Phage
NGO_1108	-	4.074796661	1.65E-07	Phage
NGO_1113	-	1.389691389	0.040021382	Phage
NGO_1114	-	2.618305649	0.049335813	Phage
NGO_1120	-	4.660642365	3.86E-08	Phage
NGO_1123	-	5.909864848	2.56E-10	Phage
NGO_1124	-	9.305526952	1.32E-08	Phage
NGO_1125	-	8.768899469	2.97E-07	Phage
NGO_1126	-	6.53561962	1.44E-13	Phage
NGO_1127	-	4.115871293	7.07E-07	Phage
NGO_1128	-	8.483568539	7.54E-14	Phage
NGO_1129	-	10.51281954	8.23E-12	Phage
NGO_1130	-	5.119357578	1.12E-10	Phage
NGO_1131	-	1.438123416	0.047498576	Phage
NGO_1144	-	-3.124762869	0.016782554	Phage
NGO_1610.1	-	5.592310493	1.43E-11	Phage
NGO_1614	-	5.610846628	2.74E-12	Phage
NGO_1615	-	5.663532551	1.38E-12	Phage
NGO_1616	-	4.306225194	1.35E-08	Phage
NGO_1617	-	7.343862263	0.000290613	Phage
NGO_1618	-	5.361734991	6.74E-10	Phage
NGO_1619	-	5.38354324	6.97E-09	Phage
NGO_1620	-	6.049033592	6.44E-08	Phage
NGO_1623	-	6.513481739	7.12E-13	Phage
NGO_1624	-	6.126721757	1.55E-12	Phage
NGO_1630	-	-4.564044188	7.85E-09	Phage
NGO_1631	-	6.508297179	2.89E-10	Phage
NGO_1633	-	8.33941502	2.94E-06	Phage
NGO_1634	-	5.557412154	1.38E-10	Phage
NGO_1635	-	4.995578192	3.33E-09	Phage
NGO_1636	-	4.491405779	3.23E-08	Phage
NGO_1638	-	5.340363232	6.18E-11	Phage
NGO_1640	RusA	3.658394177	3.25E-06	Phage

**CRISPRi-*ngo1630;* Change in gene transcript abundance (1 mM vs. 0 mM IPTG)**				
NGO_1642	-	6.413380652	0.006149306	Phage
BCHEFKBB_00117	-	-1.503444009	0.032373922	Hypothetical
NGO_0428	-	-2.821614684	0.004729112	Hypothetical
BCHEFKBB_00564	-	-2.219263265	0.01990983	Hypothetical
NGO_0622	-	3.009199773	0.004771142	Hypothetical
NGO_0635	-	-1.695339616	0.012514837	Hypothetical
BCHEFKBB_00710	-	-2.140793042	0.020077659	Hypothetical
NGO_03735	-	-2.754834639	0.005858968	Hypothetical
BCHEFKBB_01032	-	-7.035421819	0.001006409	Hypothetical
NGO_1197	-	-1.405268494	0.036933601	Hypothetical
BCHEFKBB_01298	-	-3.897780783	0.000305695	Hypothetical
NGO_1450	-	1.736123565	0.010091176	Hypothetical
NGO_1488	-	-1.478813398	0.029021757	Hypothetical
BCHEFKBB_01560	-	3.333480005	0.004729112	Hypothetical
NGO_08305	-	1.737339453	0.029312361	Hypothetical
BCHEFKBB_01699	-	-4.260602841	1.83E-07	Hypothetical
NGO_1760	-	-1.69978596	0.016228845	Hypothetical
NGO_09430	-	-6.814861183	0.002863891	Hypothetical
NGO_1847	-	-3.643595925	7.54E-07	Hypothetical
NGO_2026	-	-1.640219946	0.025646387	Hypothetical
BCHEFKBB_00695	-	-2.684812788	0.007289357	Membrane
NGO_1063	SliC	2.635101361	0.000147354	Membrane
NGO_1972	MafA3	1.364198923	0.044489447	Membrane
BCHEFKBB_01056	-	-1.3463745	0.048175574	Membrane
NGO_08230	OpaH	-3.183541214	7.30E-06	Membrane
NGO_2012	GlnP	-1.469044178	0.029183571	Membrane
NGO_2050	EfeO	-2.466789924	0.000396101	Membrane
NGO_2137	MetN	2.387450211	0.000528697	Membrane
NGO_01470	-	1.43790725	0.039430347	Metabolism
BCHEFKBB_00813	CysI	1.5910253	0.03035342	Metabolism
BCHEFKBB_01171	YigL	-2.574850168	0.001396545	Metabolism
NGO_1227	AmpA	-1.350978317	0.042152453	Metabolism
NGO_1292	DsbB	1.471001995	0.027683205	Metabolism
BCHEFKBB_01573	-	7.519714955	0.000125561	Metabolism
NGO_1721	-	1.333929205	0.044975736	Metabolism
BCHEFKBB_01957	YigZ	-1.428000892	0.032097485	Metabolism
NGO_2051	-	2.146843791	0.002141044	Metabolism
NGO_2080	-	4.181692025	1.92E-08	Metabolism
NGO_0025	MpeR	2.738148884	0.003490786	Transcription
NGO_0990	-	2.024176444	0.003085258	Transcription
NGO_08295	-	-3.096145223	0.000600932	Transposase
NGO_02015		-2.478211188	0.001462357	ROS
NGO_0114	Glr3	1.404353465	0.035759496	ROS
NGO_07995	-	1.534336724	0.02870959	Pilus

**CRISPRi-*ngo1630*; Change in gene transcript abundance (1 mM vs. 0 mM IPTG)**				
BCHEFKBB_01906	-	-1.955181839	0.011618237	Restriction Modification
NGO_1967	-	-1.460739922	0.03340408	Toxin-Antitoxin

1. If NGO number not assigned, locus tag assigned instead

2. Based on predicted or known functions

Supplemental Table 4. Most prophage genes that display a difference in transcript abundance are induced upon *ngo0479*, *ngo1116*, and *ngo1630* repression. List of prophage genes present in NGOϕ1 (A), NGOϕ2 (B), and NGOϕ3 (C) that were identified through RNA-sequencing to display a difference in transcript abundance in CRISPRi-*ngo0479*,-*ngo1116*, and -*ngo1630* strains treated with versus without 1 mM IPTG. Values indicate the Log2Fc, and colors indicate the direction and magnitude of the transcript change. Genes displaying an * denote that the change in transcript abundance could be from that gene and/or in a paralogous gene.

4A

Ngoϕ1: 1mM IPTG vs. 0 mM IPTG				
**Phage Gene**	**CRISPRi-*ngo0479***	**CRISPRi-*ngo1116***	**CRISPRi-*ngo1630***	**Potential Paralog Hit**
NGO0463	2.5			
NGO0464	1.9			
NGO0465	3.2		2.8*	*ngo1616* and/or *ngo1130*
NGO0467	2.2*		3.0*	*ngo1618* and/or *ngo1128*
NGO0468			2.9*	*ngo1619* and/or *ngo1127*
NGO0469		6.0*	10*	*ngo1620* and/or *ngo1126*
NGO0470			6.7*	ngo1621 and/or *ngo1125*
NGO0472	5.0		2.3*	ngo1623 and/or ngo1123
NGO0474	3.4*	4.8*		*ngo1121*
NGO0479	-3.6			
NGO0479.1	3.1			
NGO0484		2.3*		*ngo1111*
NGO0485	2.0	1.6		
NGO0486		6.5*		*ngo1109*
NGO0487	1.7*	2.6*	2.0*	*ngo1638* and/or *ngo1108*
NGO0489	2.0			
NGO0490	1.7			
NGO0497		-2.1		
NGO0498		2.2		
NGO0503	1.9			
NGO0504		6.8		
NGO0506	4.3			
**Total**	**14**	**9**	**7**	

4B

Ngoϕ2: 1mM IPTG vs. 0 mM IPTG				
**Phage Gene**	**CRISPRi-*ngo0479***	**CRISPRi-*ngo1116***	**CRISPRi-*ngo1630***	**Potential Paralog Hit**
NGO1093	3.5			
NGO1094	1.7			
NGO1098		1.6		
NGO1101		1.5		
NGO1102		4.2		
NGO1104		4.2		
NGO1105	3.4*	3.9*	3.8*	*ngo1640*
NGO1108		2.8*	4.1*	*ngo1638* and/or *ngo0488*
NGO1109		6.9*		*ngo0486*
NGO1110		4.3		
NGO1111		2.6*		*ngo0484*
NGO1113			1.4*	*ngo1632* and/or *ngo0480*
NGO1114		2.0*	2.6*	*ngo1631* and/or *ngo0481*
NGO1116		-4.6		
NGO1119	-2.8			
NGO1120			4.7	
NGO1121	5.3*	4.5*		*ngo0474*
NGO1123			5.9*	*ngo1623* and/or *ngo0472*
NGO1124	-5.8*		9.3*	*ngo1622* and/or *ngo0471*
NGO1125			8.8	
NGO1126	3.0*		6.5*	*ngo1620* and/or *ngo0469*
NGO1127			4.1*	*ngo1619* and/or *ngo0468*
NGO1128		2.2	8.5	
NGO1129		2.6*	11*	*ngo1617* and/or *ngo0466*
NGO1130		1.4*	5.1*	*ngo1616* and/or *ngo0465*
NGO1131	-4.0*	2.3*	1.4*	
NGO1132		3.7		
NGO1137		2.5*		*ngo1641*
NGO1141	7.0	8.7		
NGO1143	6.3			
NGO1144			-3.1	
NGO1145	3.1			
**Total**	**11**	**19**	**15**	

4C

Ngoφ3: 1mM IPTG vs. 0 mM IPTG				
**Phage Gene**	**CRISPRi-*ngo0479***	**CRISPRi-*ngo1116***	**CRISPRi-*ngo1630***	**Potential Paralog Hit**
NGO1610.1		-6.6	5.6	
NGO1614			5.6	
NGO1615			5.7	
NGO1616		1.7*	4.3*	*ngo1130* and/or *ngo0465*
NGO1617			7.3*	*ngo1129* and/or *ngo0466*
NGO1618		4.4*	5.4*	*ngo1128* and/or *ngo0467*
NGO1619		2.4*	5.4*	*ngo1127* and/or *ngo0468*
NGO1620	6.2*		6.0*	*ngo1126* and/or *ngo0469*
NGO1622		-6.7*		*ngo1124* and/or *ngo0471*
NGO1623		2.4*	6.5*	*ngo1123* and/or *ngo0472*
NGO1624		-6.6	6.1*	*ngo1120* and/or *ngo0475*
NGO1630			-4.6	
NGO1631			6.5*	*ngo1114* and/or *ngo0480*
NGO1633			8.3	
NGO1634			5.6	
NGO1635			5.0	
NGO1636			4.5	
NGO1638		1.5*	5.3*	*ngo1108* and/or *ngo0487*
NGO1640			3.7*	
NGO1641	2.8			
NGO1642			6.4*	*ngo1137.1*
NGO1645	2.8*	3.4*		*ngo1140*
**Total**	**3**	**9**	**19**	
